# Dietary Polyphenols as Prospective Natural-Compound Depression Treatment from the Perspective of Intestinal Microbiota Regulation

**DOI:** 10.3390/molecules27217637

**Published:** 2022-11-07

**Authors:** Xuanpeng Wang, Jing Yu, Xin Zhang

**Affiliations:** 1 Guangdong Qingyunshan Pharmaceutical Co., Ltd., Shaoguan 512699, China; 2 Department of Food Science and Engineering, Ningbo University, Ningbo 315211, China

**Keywords:** dietary polyphenols, intestinal microbiota, depression

## Abstract

The broad beneficial effects of dietary polyphenols on human health have been confirmed. Current studies have shown that dietary polyphenols are important for maintaining the homeostasis of the intestinal microenvironment. Moreover, the corresponding metabolites of dietary polyphenols can effectively regulate intestinal micro-ecology and promote human health. Although the pathogenesis of depression has not been fully studied, it has been demonstrated that dysfunction of the microbiota-gut-brain axis may be its main pathological basis. This review discusses the interaction between dietary polyphenols and intestinal microbiota to allow us to better assess the potential preventive effects of dietary polyphenols on depression by modulating the host gut microbiota.

## 1. Introduction

Polyphenols are the most abundant natural compounds in plants and can be found in fruits, vegetables, tea, coffee, cocoa, and wine, among other things [1]. Polyphenols are classified as flavonoids or non-flavonoids based on the presence of one or more hydroxyl groups attached to their benzene ring. Flavonoids share a carbon skeleton with diphenyl propane, which has two benzene rings joined by a three-carbon chain. The middle three-carbon chain joins the A-benzene ring to form a closed pyran ring. Flavonoids involve the common carbon skeleton of diphenylpropane, in which two benzene rings are connected by a linear three-carbon chain. The central three-carbon chain forms a closed pyran ring with the A-benzene ring. Flavonoids are classified as flavones, flavonoids, anthocyanins, flavanones, flavonols, and isoflavones based on the oxidation state of the core pyran ring. The main types of nonflavonoids are phenolic acids, which can be subdivided into benzoic acid derivatives, such as gallic acid and protocatechuic acid, and cinnamic acid derivatives, including coumaric acid, caffeic acid, and ferulic acid. Dietary polyphenols refer to phenolic substances obtained from natural sources [2]. Not only do dietary polyphenols have antioxidant properties, but they are emerging as compounds with antidepressant efficacy [3]. As a flavonoid, hesperidin is found to have a high content in citrus fruits [4]. Studies have shown that hesperidin can inhibit apoptosis and protect neuronal degeneration by increasing the levels of PI3K, Akt, and mTOR [5]. Apigenin is widely distributed in warm tropical vegetables and fruits, especially in celery. By inhibiting p38 and JNK, apigenin can pass the blood-brain barrier (BBB) and have an anti-inflammatory impact on BV-2 and primary microglia [6].

Trillions of bacteria engage in complicated interactions with the host system in the human gut microbiota [7] and human genetic and metabolic diversity have also been found in the gut microbiota [8]. In addition, important homeostasis consequences result from the stability of the gut microflora. The host’s immune system and general health are maintained by the gut microbiota, which is also engaged in the management of nutrients and several metabolic pathways (such as bile acid metabolism, choline metabolism, and tryptophan metabolism for different homeostatic regulation) [9]. Regarding its low bioavailability, curcumin directly absorbed by the small intestine represents only a small part, the majority remains in the intestinal tract [10]. Several strategies aiming to improve its oral bioavailability have been considered [11]. In the gut, curcumin is biologically converted to metabolites by the microbiota, those metabolites can in turn regulate the composition and function of intestinal microbiota [12]. Reduced immune function can arise from intestinal mucosal barrier injury caused by intestinal microflora homeostasis disruption [13]. Numerous illnesses, including depression, multiple sclerosis, diabetes, autism, and cancer are linked to disturbed gut flora [14]. Through the neurological, endocrine, immunological, and metabolic systems, the brain’s interactions with the gut microbiota mostly have an indirect impact on cognition [15], sleep [16], and mood [17]. The gut microbiota not only mediates the physiological processes of host metabolism and immunity but also plays a significant role in the bidirectional response of the gastrointestinal tract and the central nervous system [18], according to numerous animal and clinical studies conducted over the past ten years [19].

Depression is one of the most common mental illnesses, with continuous and long-term depression as the main clinical feature, and is the most important type of preventable mental illness [20]. Depression currently affects 4.4% (322 million people) of the population [21]. In fact, according to the World Health Organization (WHO), depression will overtake physical infirmity as the second-leading cause of mental disease in the future [22]. It is quite difficult to understand how depression develops. The monoamine theory, one of the most widely recognized theories, contends that depression is brought on by lower levels of monoamines, such as gamma-aminobutyric acid (GABA), norepinephrine (NE), and serotonin (5-HT) in the cranial nervous system [23].

Increased oxidative stress and elevated inflammatory markers can trigger depressive symptoms [24]. According to randomized controlled research, antioxidant supplementation for 6 weeks significantly raised plasma antioxidant levels in depressed individuals and was linked to a considerable decrease in depressive symptoms [25]. Dietary polyphenols are a class of antioxidants that appear in a variety of antioxidant supplements and offer a range of physiological advantages that aid in the treatment of mental diseases. The most typical form of depression, major depressive disorder (MDD), is a serious and incapacitating mental condition [26]. MDD poses a serious challenge to health systems because it frequently recurs and is difficult to treat [27]. The pathophysiology of MDD has not yet been determined, but an increasing number of animal and clinical research have demonstrated that the “microbiota-gut-brain” axis’ malfunction is the primary pathogenic cause of depression and that it may also have potential influencing variables [28]. Through the gut-brain axis (GBA), bidirectional interactions between the central nervous system (CNS) and the gastrointestinal tract have been known to affect mood. Studies have shown that gastrointestinal diseases often accompany MDD, and the behavior and diet of MDD patients can change the composition of gut microbiota and have an impact on the pathogenesis of MDD [29].

Polyphenols can regulate intestinal flora and maintain intestinal stability [30]. Naturally derived polyphenols, such as quercetin, can reduce depressive and anxious behaviors in rats [31]. By modulating the short-chain fatty acids (SCFAs) produced by the gut microbiota, dietary polyphenols affect the levels of neurotransmitters in the brain, the development of the central nervous system, and immune barriers. They also treat depression by lowering the stress-induced increases in brain cortisol through the vagus nerve [32]. Although it has been suggested that dietary polyphenols have a role in controlling gut flora, nothing is known about how these two organisms interact. To offer fresh perspectives on the prevention and treatment of depression with dietary polyphenols, this review will discuss the interaction between dietary polyphenols and intestinal microflora, with a focus on how dietary polyphenols regulate intestinal flora through GBA and affect the development of depression.

## 2. The Metabolism of Dietary Polyphenols in the Gut

**Figure 1:**
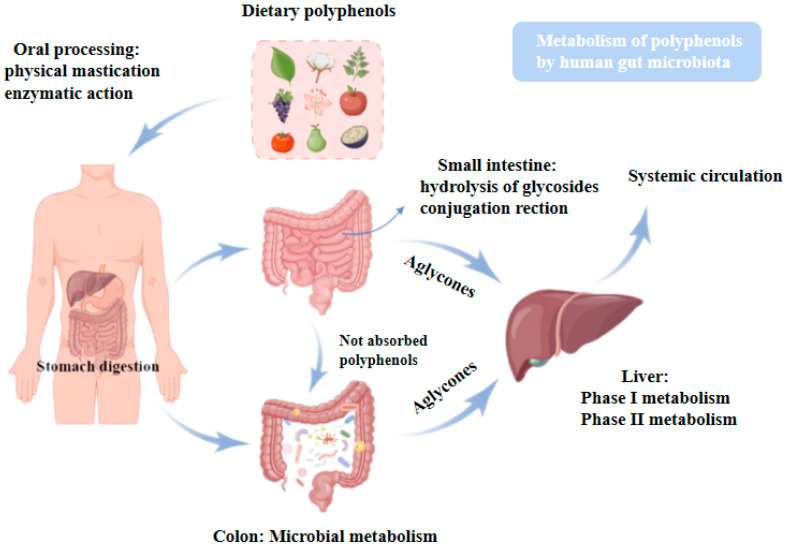
The metabolism of polyphenols by the human gut microbiota.

When dietary polyphenols are ingested, they undergo a long journey through the gastrointestinal tract (Figure 1). Biotransformation of polyphenols occurs in the enterocytes of the small and large intestines [33]. By brush border or microbial enzymes, many polyphenols are hydrolyzed in the small intestine, and the resulting aglycone compounds are typically absorbed by enterocytes by passive diffusion. Unabsorbed polyphenols are broken down into smaller phenolic compounds in the large intestine, and the microbiota breaks down glycosidic linkages and disassembles polyphenols’ biphenylpropane structure [34]. Aglycones and oligomers are mostly released by microbial esterases and glycosidases during the degradation of polyphenols [35]. For example, the hydrolysis products of ester bonds of catechin gallates, such as allocated polyphenols (-)-epigallocatechin-3-gallate (EGCG) and (-)-epicatechin-3-gallate (ECG), in the gut undergo phase II biotransformation in the gut and liver and interact with the gut microbiota to release free catechins, glucaldehyde acidified/sulfated/methylated conjugates, phenolic acids, and other catabolites [36]. Anthocyanins are deglycosylated and converted to phenolic acids such as protocatechuic acid, syringic acid, and gallic acid by colonic bacteria [37]. Protocatechuic acid, the primary metabolite of anthocyanins, is a physiologically active chemical with significant promise in treating a variety of chronic diseases [38]. Overall, this extensive microbial metabolism ultimately breaks down dietary polyphenols into a limited number of simple aromatic metabolites.

## 3. Effects of Dietary Polyphenols on the Intestinal Microbial Environment

### 3.1. The Effect of Dietary Polyphenols on the Enzymatic Activity of Gut Microbiota

Intestinal enzyme activity is critical for the digestion and absorption of animal nutrients, as well as for body growth and development. The intestinal microbiota has a diverse spectrum of enzyme systems that are engaged in a variety of physiological activities, such as the movement of energy, materials, and genetic information of the host [39], and mostly contains hydrolases, oxidoreductases, lyases, and transferases. Enzymatic mechanisms in the gut microbiota digest and absorb 90–95% of polyphenols, which are then transformed into low molecular weight bioactive metabolites [40]. According to research, inhibiting the activities of α-amylase, α-glucosidase, and β-glucosidase decreases oxidative stress and inflammation-related hyperglycemia while also modifying the gut microbiota to lower blood sugar levels [41]. Anthocyanins derived from blueberries and blue honeysuckle can be developed as possible α-glucosidase inhibitors [42], delaying carbohydrate digestion and extending digestion time, resulting in a decreased rate of glucose absorption, and therefore slowing the digestion of the meal [43]. Currently, dipeptidyl peptidase IV inhibition has been recognized as an effective strategy for the management of type 2 diabetes by enhancing the incretin system, thereby promoting beta-cell efficiency and insulin release in a glucose-dependent manner [44]. The quercetin contained in mugwort extract has a strong inhibitory effect on dipeptidyl peptidase IV, and the maximum inhibition rate of dipeptidyl peptidase IV is 90% when the extract concentration is 4000 μg/mL [45]. Tea polyphenols, one of the most common dietary polyphenols, have been found to influence the enzymatic activity of the gut microbiota [46]. Tea polyphenols were shown to significantly reduce α-glucosidase levels in rats fed high-fat diets, and they also helped to ameliorate hyperglycemia symptoms in obese rats [47]. Tea polyphenols can directly affect some intestinal microbial enzymes, primarily by interacting with enzyme protein molecules [48]. According to the study, catechin can bind to the ATP-binding site on the gyrase B subunit, blocking the *Escherichia coli* DNA gyrase from activating [49].

### 3.2. Effects of Dietary Polyphenols on Gut Microflora

While polyphenols undergo a series of metabolisms in the gut, polyphenols also shape the microbiota and have a positive impact on health [50]. Dietary polyphenols can change the variety and composition of the gut microbiota and can also modify the quantities of intestinal metabolites, such SCFAs and bile acids [51]. Polyphenols are not only beneficial in improving local damage in the intestine, such as intestinal inflammation and permeability, but also in preventing or treating some systemic metabolic diseases, such as diabetes and obesity [52]. After entering the circulation, dietary polyphenols and their metabolites may have some local biological effects in the gut, such as protecting the gut barrier, as well as some systemic effects [53]. Specific bacterial populations in the gut are impacted by a diet high in polyphenols. By encouraging the development of lactic acid bacteria, such as *Lactobacillus* and *Bifidobacterium*, polyphenols have prebiotic effects (Figure 1). These prebiotics can also effectively control the microorganisms *Faecalibacterium prausnitzii* and *Akkermansia muciniphila*, which have anti-obesity characteristics [54]. Theanine and flavonoid glycosides and catechins found in black tea increase the formation of bifidobacterial. Polyphenols not only encourage the growth of helpful bacteria in the gut, but they also prevent the growth of potentially harmful bacteria [55]. Anthocyanins have been shown to limit the development of Gram-positive bacteria (*Bacillus subtilis*, *and Enterococcus faecalis*) and Gram-negative bacteria (*Escherichia coli, Citrobacter freundii, Pseudomonas aeruginosa*) [56]. Citrus fruits include flavonoids (such as hesperidin and naringenin) that can inhibit *Escherichia coli*, *Staphylococcus aureus,* and *Salmonella typhimurium* [57]. As a flavonoid, quercetin has antioxidant, anticancer, and neuroprotective effects [58]. In a rat in vivo dietary intervention study, gavage of rats with quercetin reduced the *Firmicutes*/*Bacteroidetes* ratio and inhibited the growth of *Erysipelas* and *Bacillus*. Studies of dietary intervention experiments and metagenomic sequencing in mice have shown that changes in *Firmicutes*/*Bacteroidetes* ratios are strongly associated with diseases such as obesity. A different animal study found that mice’s intestinal microbiota was altered by fermented green tea extract high in tea polyphenols, affecting the phyla *Firmicutes* and *Bacteroidetes* and the ratio of *Bacteroidetes* to *Prevotella* [59].

## 4. The Effect of Gut Microbiota on Depression

### 4.1. Gut Microbiota and GBA

**Figure 2:**
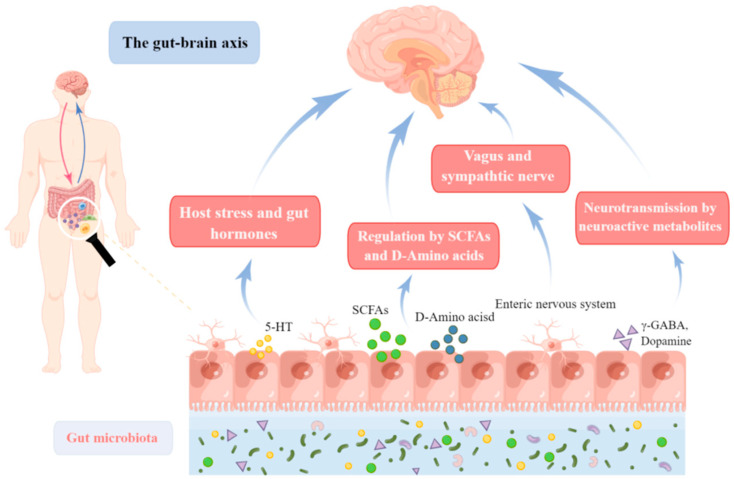
The role of the GBA in depression. Dysbiosis in the gut flora can be caused by an unhealthy lifestyle, excessive and continuing stress, disease, or other factors. The GBA’s bidirectional control of aberrant physiological states via neural, immunological, or chemical signals may result in depression.

Depression is a significant mental condition that has been linked to GBA and gut microbiota. This indicates that alterations in gut microbiota and GBA are significant pathways for elucidating the pathophysiology of depression and foretelling potential depression treatments [60]. In fact, the number of bacteria in the gut is many times greater than that of human cells, and the number of related genes is 100 times greater than that of the human genome [61]. As a result, the microbiota in the gut is the most diverse and abundant in the human body. In addition to preventing pathogen invasion, increasing digestion and metabolism, boosting nutrient absorption, and controlling the development and operation of the host immune system, the gut microbiota is crucial to many physiological processes in the human body [62]. As a result, the hypothalamic-pituitary-adrenal (HPA) axis, immunity, and neurotransmitters between the gastrointestinal system and the brain are all integrated by the gastrointestinal-brain axis (GBA) [63]. Its malfunction or imbalance is linked to a number of immunological, mental, and neurological illnesses [64] (Figure 2).

The CNS can influence the gut microbiota both directly and indirectly [65]. For example, the brain’s HPA axis and autonomic nervous system have a direct influence on gut physiology. Similarly, the CNS influences the composition and function of gut microorganisms indirectly by producing signaling molecules such as cytokines and antimicrobial peptides. The gut flora also has an impact on CNS function. Through interactions with the vagus nerve and enteric nervous system, gut bacteria can influence CNS development and regulation. The immune system is one of the primary physiological systems that the gut microbiota regulates in depressive-related pathways. Changes in gut flora boost peripheral immunity, resulting in an inflammatory response [66]. When diverse inflammatory chemicals reach the CNS via various pathways, the activation of microglia, a prominent source of pro-inflammatory molecules in the brain, increases the likelihood of depression [67]. To control the bacterial population in the gut, the gut environment, the central nervous system, and the immune system all collaborate [68]. For example, it has been shown that the mucosal antimicrobial peptide Reg gene family member 3γ (RegIIIγ), which is secreted by intestinal epithelial cells, binds to the peptidoglycan on the surface of Gram-positive bacteria and directly kills them [69].

GBA regulation is influenced by neurotransmitters and neurotrophic factors [70]. The gut microbiota has been demonstrated in studies to be involved in the creation of numerous neuroactive chemicals such as melatonin, GABA, catecholamines, acetylcholine, and histamine [71]. Disruptions in the gut microbiota can induce decreased neurotransmitter and other neurotrophic factor syntheses, which can change mood and body movement and increase the risk of depression. For example, GABA signaling dysregulation has been associated with depression [72]. GABA is generated by a variety of bacteria, including *Bifidobacterium* and *Lactobacillus* [73]. *Lactobacillus rhamnosus JB-1* (a common *Lactobacillus* species) was also reported to reduce anxiety and depressive behavior in mice in a vagus-dependent manner and to produce GABA. GABA is produced by *Bifidobacterium* by enzymatic dehydration of rat glutamate.

### 4.2. Changes in Gut Microbiota in Depressed Patients

The research of genetic, neurochemical, and environmental factors is critical for depression therapy [74]. Bidirectional interactions between neurotransmitters in the brain and the central nervous system, enteric nervous system, and gastrointestinal tract demonstrate the effects of these systems on emotion, pain and stress regulation, and brain function [75]. This shows that the composition and changes in the gut microbiota might affect and interfere with the mental health of depressed persons [76]. Animal studies have revealed that the gut microbiota may greatly influence host behavior [77], primarily via neurotransmission, the HPA axis, and inflammation. Furthermore, the presence or exposure to pathogenic bacteria in the stomach increases depressive-like behaviors [78]. Because of their ability to produce exotoxins and generate settings conducive to inflammation, this species’ overgrowth may exacerbate depressive symptoms. According to clinical investigations, the incidence of gastrointestinal illnesses in persons with depression is around 29.6% [79]. Additional research has revealed that depressed people have drastically altered gut microbiota [80]. For instance, a study comparing the gut microbiota of 46 depressed patients with that of 30 in the control group revealed that the abundance of *Bacteroidetes*, *Proteobacteria*, and *Actinobacteria* was significantly higher in the depressed patients than it was in the control group, while the abundance of Firmicutes decreased quickly [81]. *Prevotella* and *Klebsiella* were found in significantly higher numbers in people with major depressive disorder, according to a different study [82]. When rats with low levels of microbiota received fecal microbiota from depressed people, the transplanted rats began to exhibit depressive symptoms.

### 4.3. The Interactions of SCFAs and Gut Microbiota on Depression

Specifically, through direct changes in critical metabolite levels and indirect impacts of circulating serum metabolite changes, metabolism is a primary avenue by which the gut microbiota affects depression through the GBA. These effects further affect alterations in the CNS that control depressive behavior. The important metabolites created by the typical microbiota play a direct or indirect role in maintaining healthy bodily functions as well as controlling mental and emotional processes. The gut microbiota’s “hidden weapons” are SCFAs. They not only take part in energy metabolism but also control how the gut produces hormones and how different nutrients are absorbed [83]. Butyric acid is one of the most important SCFAs, it is a major fuel source for colon cells and plays a non-negligible role in gut health [84]. Butyric acid is the main nutrient of human intestinal epithelial cells; more than 95% of the butyric acid in the human body is produced and absorbed in the colon [85], and a certain level of butyric acid can keep colon cells stable, thereby preventing or inhibiting cancer, regulating intestinal flora imbalance and treating irritable bowel syndrome, antibiotic-associated enteritis, acute and chronic diarrhea, and other diseases [86]. SCFAs (e.g., acetate, propionate, and butyrate) also have potential therapeutic effects on depression [87]. Multiple SCFA-producing bacteria, including those from the genera *Subdoligranulum, Dialister, Fuscatenibacter, Ruminococcus,* and *Dorea,* were lost in stool samples from patients with pediatric depression, according to a study that compared the distal gut microbiota composition of 70 healthy and 101 depressed children [88]. Due to their role in maintaining the homeostasis of colonic regulatory T-cell populations, SCFAs primarily have immunomodulatory and anti-inflammatory actions [89]. A lack of SCFAs in the gut weakens the gut wall, allowing gut bacteria to pass through the leaky gut and causing abnormal host behavior by activating the immune system [90,91].

### 4.4. The Effect of the Antioxidant Properties of Probiotics on Depression

Antioxidant supplements have been shown to ease mood disorders. In fact, some antidepressants (e.g., escitalopram and olanzapine) are protective against oxidative stress. Probiotics exhibit antioxidant properties, and probiotics can improve depression and anxiolytic behaviors caused by associated comorbidities [92]. Probiotics’ antioxidant benefits have been related to studies on aging, diabetes, brain injury, neurodegenerative illnesses, and other disease models that all contribute to MDD and the decline of cognitive function [93]. According to research on animals, the hippocampus, cortex, and striatum of mice treated with *Enterococcus faecalis CFR3003* displayed increased activity in antioxidant enzymes such as catalase and lowered levels of oxidative stress (ROS) indicators in brain tissue [94]. Rats’ behavioral cognition and depression symptoms were improved by a probiotic supplement including *Lactobacillus rhamnosus, Lactobacillus reuteri,* and *Bifidobacterium* [95]. Researchers have also supplied individuals with type 2 diabetes with *fructooligosaccharides* (a prebiotic substance), *Lactobacillus acidophilus, Lactobacillus casei, Lactobacillus rhamnosus, Lactobacillus bulgaricus, Bifidobacterium breve, Bifidobacterium longum,* and *Streptococcus thermophilus*. This probiotic mixture lowered the quantities of superoxide and hydroxyl radicals, improved diabetes, and strengthened antioxidant defenses, including plasma glutathione levels [96]. Type 2 diabetes is intimately linked to depression and cognitive decline, despite the fact that it is not a neurodegenerative disease in and of itself. Anxiety, depression, and other mental diseases can be brought on by type 2 diabetes.

Although there are various communication pathways between the stomach and the brain, the role of gut bacteria in the development of brain illnesses is as yet unknown. We still need to learn more about the GBA and how it is impacted by gut microbiota in order to uncover new targets for the prevention and treatment of brain diseases.

## 5. Preventive and Therapeutic Effects of Dietary Polyphenols on Depression by Regulating Intestinal Microbiota

### 5.1. Dietary Polyphenols Exert Anti-Depressant Effects by Modulating Intestinal Microbiota

Oriental medicine uses *Licgusticum.* L., a plant that reduces inflammation and is high in ferulic acid, and a hydroxycinnamic acid that boosts *Bifidobacterium* relative abundance [97]. Free FA may be one of *Bifidobacterium*’s fermentation substrates, according to in vitro experiments that showed the bacteria can change it [98].

On the one hand, *Bifidobacterium* exhibits antidepressant potential through a 5-hydroxytryptophan (5-HTP)-dependent mechanism, which acts as a precursor of 5-HT in humans [99]. Several double-blind studies have shown that 5-HTP can increase the concentration of serotonin in the brain and improve depression [100]. On the other hand, after treating mice with *Bifidobacterium*, it was found that another pathway associated with depression is the glutamatergic synapse. Glutamate is an excitatory neurotransmitter in the CNS [24], glutamate transport provides a new therapeutic site for depression by activating *N*- The methyl-d-aspartate receptor (NMDAR) involved in the regulation of synaptic activity, brain plasticity, and energy reserve, thereby exerting an antidepressant effect [101].

### 5.2. Dietary Polyphenols Improve Depression by Modulating Tryptophan through Intestinal Microbiota

An essential amino acid called tryptophan is converted along the kynurenine pathway to produce a number of metabolites that are crucial to understanding the pathophysiology of depression. According to research conducted on animals, tryptophan metabolism is impacted by the modulation of polyphenol signaling and their metabolites through the kynurenine pathway [102]. Resveratrol, a natural polyphenol, was found to significantly reduce tryptophan levels and increase the ratio of kynurenine to tryptophan by 1.30 times after injecting resveratrol into healthy volunteers [103]. Black tea catechins, in particular, raised kynurenine levels in healthy volunteers, leading to a greater kynurenine-to-tryptophan ratio [104]. Researchers have also transplanted fecal microbiota from depressed patients into germ-free rats and found that they caused changes in tryptophan metabolism, anhedonia, and anxiety-like behaviors [105]. At the same time, tryptophan is utilized by the gut flora to synthesize serotonin locally, and the production of serotonin directly links the gut with nerve signaling [106].

One of the most potent blood indicators of serious depression is the depletion of serotonin, a crucial neurotransmitter in the GBA [107]. Serotonin is neuroactive and is generated peripherally, which is important for neuropsychiatric diseases such as depression [108].

### 5.3. Microbial-Derived Polyphenol Metabolites Treat Depression by Inhibiting Microglial Activation

Microglia are important immunological mediators in the CNS, and their activation is linked to clinical psychiatric symptoms and neuroinflammation [109]. Clinical investigations have demonstrated that the majority of depressed individuals exhibit an overactivation of microglia, and depression has been defined as a disorder linked to microglia [110]. Researchers have also found that gut microbiota can influence microglia dynamics, with clear differences in the microglia transcriptome between germ-free and specific pathogen-free mice [111]. Many genes involved in cell activation were down-regulated in the microglia of germ-free animals which points to the relevance of the microbiota in influencing microglial responses [112]. Dietary polyphenols used to modulate microglial activation require gut microbiota activity to produce appropriate bioactive metabolites for the treatment of depressive symptoms. The ellagitannin-like polyphenols in pomegranate extract are poorly absorbed in the small intestine and have low bioavailability [113], and, upon reaching the colon, are biotransformed by the gut microbiota to produce the bioactive compound urolithin (*6H-dibenzo[b, d]pyran-6-one derivatives*). Urolithin reduces microgliosis and amyloid-beta plaque deposition [114], reduces anxiety-like behaviors, and improves memory [115]. In primary cultures of neuronal glia, the physiologically active microbial-derived metabolite EGCG was found to prevent LPS-induced microglial activation [116].

### 5.4. Gut Microbiota and Related Polyphenol Metabolites Modulate Inflammasome Activation in the Treatment of Depression

The gut microbiota reduces the integrity of the BBB by inducing peripheral inflammation, leading to inflammasome activation which leads to a range of depressive symptoms while disrupting the composition of the gut microbiota. This is known as the microbiota-inflammasome hypothesis of major depressive disorder [117]. Inflammation is the main sign of cancer development and progression [118]. It has been found that there is a close relationship between inflammation and tumorigenesis, including proliferation, invasion, and metastasis [119]. The NLRP3 inflammasome is a key component of the innate immune system [120] and mediates caspase-1 activation and secretion of the proinflammatory cytokine IL-1β/IL-18 [121] in response to microbial infection and cell damage [122]. Therefore, NLRP3 inflammatory corpuscles play a major role in regulating inflammatory response and tumors by interfering with other cell compartments [123]. Animal experiments have proved that during high-fat diet feeding, the activation of NLRP3 inflammatory bodies may produce a low-grade systemic inflammation, thus promoting the development of colorectal cancer (CAC) [124]. This is related to NLRP3’s ability to sense the danger signals caused by a high-fat diet and promote obesity and insulin resistance caused by inflammation [125]. Various microbial pathogens that can activate the NLRP3 inflammasome have been identified, including *Salmonella typhimurium*, *Escherichia coli*, etc. [126]. Currently, the activation of this mechanism remains to be studied. Gut bacteria can activate the inflammasome directly or indirectly. In one study, Enterobacteriaceae were shown to stimulate IL-1β release via inflammasome signaling after spinal cord injury [127]. These selective members of the gut microbiota can stimulate newly recruited monocytes to induce NLRP3-dependent IL-1β release, promoting intestinal inflammation [128]. NLRP3 gene expression was elevated in human peripheral blood mononuclear cells (PBMCs) in patients with depression and serum IL-1β and IL-18 levels were also elevated [129]. Dietary polyphenol intake can reduce inflammasome activation [130] and alleviate depressive symptoms. The components in lychee seed are mostly polyphenols [131], such as rutin, quercetin, catechin, and procyanidins [132]. The latest research shows that lychee seed polyphenol (LSP) can induce autophagy through the LRP1/AMPK pathway and significantly inhibit NLRP3 inflammatory bodies [133]. EGCG has been shown to affect inflammasome signaling in multiple models [134]. Compared with an induced renal failure model, EGCG down-regulates NLPR3 gene expression through a pathway involved in the inflammatory regulator heme oxygenase-1 [135], and NLRP3 gene knockout can reduce depression-like behavior in mice due to chronic stress.

## 6. Conclusions

The GBA functions as a bidirectional neuroendocrine system, linking the intestinal microbiota and the brain. The dysbiosis of the gut microbiota has an impact on the emergence of a variety of chronic disorders. Dietary polyphenols are promising compounds for the treatment of depression. They can maintain the intestinal microenvironment’s homeostasis, and their metabolites can effectively regulate intestinal micro-ecology. However, more clinical studies are required to determine the intervening effects of dietary polyphenols and their metabolites on depression.
